# 4-Hy­droxy-*N*′-(4-hy­droxy-3-nitro­benzyl­idene)benzohydrazide

**DOI:** 10.1107/S1600536811000195

**Published:** 2011-01-08

**Authors:** Zhen Zhang

**Affiliations:** a Experimental Center, Linyi University, Linyi Shandong 276005, People’s Republic of China

## Abstract

The mol­ecule of the title compound, C_14_H_11_N_3_O_5_, assumes an *E* configuration with respect to the methyl­idene unit. An intra­molecular O—H⋯O hydrogen bond is present in the mol­ecule. The dihedral angle between the mean planes of the two benzene rings is 5.46 (15)°. The crystal structure is stabilized by inter­molecular O—H⋯O, O—H⋯N, and N—H⋯O hydrogen bonds.

## Related literature

For the biological applications of hydrazone compounds, see: Ajani *et al.* (2010 ▶); Avaji *et al.* (2009 ▶); Fan *et al.* (2010 ▶); Rasras *et al.* (2010 ▶). For similar hydrazone compounds, see: Ahmad *et al.* (2010 ▶); Ban (2010 ▶); Ji & Lu (2010 ▶); Shalash *et al.* (2010 ▶).
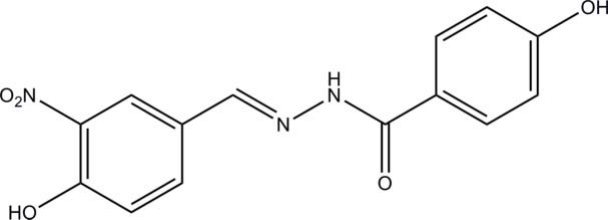


## Experimental

### 

#### Crystal data

C_14_H_11_N_3_O_5_
*M* *_r_* = 301.26
Monoclinic, 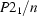

*a* = 8.786 (3) Å
*b* = 14.882 (2) Å
*c* = 10.3064 (17) Å
β = 91.100 (2)°
*V* = 1347.3 (5) Å^3^
*Z* = 4
Mo *K*α radiation
μ = 0.12 mm^−1^
*T* = 298 K
0.32 × 0.30 × 0.29 mm

#### Data collection

Bruker SMART APEX CCD area-detector diffractometer
Absorption correction: multi-scan (*SADABS*; Bruker, 2009 ▶) *T*_min_ = 0.964, *T*_max_ = 0.967
7079 measured reflections
2952 independent reflections
1353 reflections with *I* > 2σ(*I*)
*R*_int_ = 0.050

#### Refinement

*R*[*F* ^2^ > 2σ(*F* ^2^)] = 0.054
*wR*(*F* ^2^) = 0.137
*S* = 1.06
2952 reflections
204 parameters
1 restraint
H atoms treated by a mixture of independent and constrained refinement
Δρ_max_ = 0.21 e Å^−3^
Δρ_min_ = −0.21 e Å^−3^

### 

Data collection: *APEX2* (Bruker, 2009 ▶); cell refinement: *SAINT* (Bruker, 2009 ▶); data reduction: *SAINT*; program(s) used to solve structure: *SHELXS97* (Sheldrick, 2008 ▶); program(s) used to refine structure: *SHELXL97* (Sheldrick, 2008 ▶); molecular graphics: *SHELXTL* (Sheldrick, 2008 ▶); software used to prepare material for publication: *SHELXTL*.

## Supplementary Material

Crystal structure: contains datablocks global, I. DOI: 10.1107/S1600536811000195/su2243sup1.cif

Structure factors: contains datablocks I. DOI: 10.1107/S1600536811000195/su2243Isup2.hkl

Additional supplementary materials:  crystallographic information; 3D view; checkCIF report

## Figures and Tables

**Table 1:** Hydrogen-bond geometry (Å, °)

*D*—H⋯*A*	*D*—H	H⋯*A*	*D*⋯*A*	*D*—H⋯*A*
O1—H1*A*⋯O2^i^	0.82	1.93	2.736 (3)	169
O1—H1*A*⋯N2^i^	0.82	2.61	3.121 (3)	122
O3—H3⋯O4	0.82	1.90	2.592 (4)	142
N1—H1⋯O5^ii^	0.90 (1)	2.32 (1)	3.203 (4)	167 (3)

Symmetry codes: (i) 
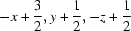
; (ii) 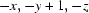.

**Fig. 1.**
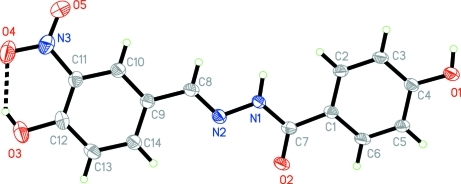
A view of the molecular structure of the title compound, showing the displacement ellipsoids at the 30% probability level. The intramolecular O—H···O hydrogen bond is shown as a dashed line.

**Fig. 2.**
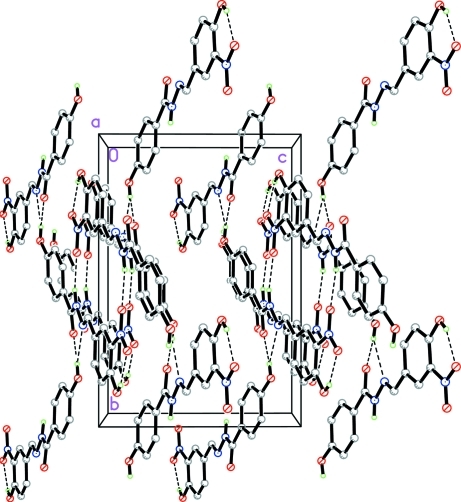
A perspective view along the a-axis of the crystal packing of the title compound. The hydrogen bonds are shown as dashed lines. H-atoms not involved in hydrogen bonding have been omitted for clarity.

## supplementary crystallographic information

### Comment

Hydrazone compounds have received much attention due to their potential applications in biological chemistry (Ajani *et al.*, 2010; Avaji *et al.*, 2009; Fan *et al.*, 2010; Rasras *et al.*, 2010). As a continuatio of our work on the hydrazone compounds, the new title hydrazone compound was prepared and structurally characterized.

The molecule of the title compound assumes an *E* configuration with respect to the methylidene unit (Fig. 1). The dihedral angle between the best mean planes of the two benzene rings is 5.46 (15)°. An intramolecular O—H···O hydrogen bond is present in the molecule (Table 1). The bond lengths are comparable to those observed in similar hydrazone compounds (Ahmad *et al.*, 2010; Ban, 2010; Ji & Lu, 2010; Shalash *et al.*, 2010).

The crystal structure is stabilized by intermolecular O—H···O, O—H···N, and N—H···O hydrogen bonds (Table 1 and Fig. 2).

### Experimental

An ethanol solution (50 ml) of 4-hydroxybenzohydrazide (0.01 mol) and 4-hydroxy-3-nitrobenzaldehyde (0.01 mol) was stirred at room temperature for 30 min to give a yellow solution. Yellow block-shaped single crystals, suitable for X-ray diffraction, were formed by slow evaporation of the solution in air.

### Refinement

The amino H-atom, H1, was located from a difference Fourier map and refined with a N—H distance restraint to 0.90 (1) Å. The remaining H atoms were positioned geometrically and refined using the riding-model approximation: C—H = 0.93 Å, and O—H = 0.82 Å, with *U*_iso_(H) = 1.2*U*_eq_(parent C-atom) and 1.5*U*_eq_(parent O-atom).

### Crystal data

**Table:**

C_14_H_11_N_3_O_5_	*F*(000) = 624
*M**_r_* = 301.26	*D*_x_ = 1.485 Mg m^−^^3^
Monoclinic, *P*2_1_/*n*	Mo *K*α radiation, λ = 0.71073 Å
*a* = 8.786 (3) Å	Cell parameters from 1674 reflections
*b* = 14.882 (2) Å	θ = 2.4–26.9°
*c* = 10.3064 (17) Å	µ = 0.12 mm^−^^1^
β = 91.100 (2)°	*T* = 298 K
*V* = 1347.3 (5) Å^3^	Block, yellow
*Z* = 4	0.32 × 0.30 × 0.29 mm

### Data collection

**Table:**

Bruker SMART APEX CCD area-detector diffractometer	2952 independent reflections
Radiation source: fine-focus sealed tube	1353 reflections with *I* > 2σ(*I*)
graphite	*R*_int_ = 0.050
ω scans	θ_max_ = 27.5°, θ_min_ = 2.4°
Absorption correction: multi-scan (*SADABS*; Bruker, 2009)	*h* = −9→11
*T*_min_ = 0.964, *T*_max_ = 0.967	*k* = −19→19
7079 measured reflections	*l* = −13→7

### Refinement

**Table:**

Refinement on *F*^2^	Primary atom site location: structure-invariant direct methods
Least-squares matrix: full	Secondary atom site location: difference Fourier map
*R*[*F*^2^ > 2σ(*F*^2^)] = 0.054	Hydrogen site location: inferred from neighbouring sites
*wR*(*F*^2^) = 0.137	H atoms treated by a mixture of independent and constrained refinement
*S* = 1.06	*w* = 1/[σ^2^(*F*_o_^2^) + (0.0262*P*)^2^ + 0.9404*P*] where *P* = (*F*_o_^2^ + 2*F*_c_^2^)/3
2952 reflections	(Δ/σ)_max_ < 0.001
204 parameters	Δρ_max_ = 0.21 e Å^−^^3^
1 restraint	Δρ_min_ = −0.21 e Å^−^^3^

### Special details

**Table:**

Geometry. All e.s.d.'s (except the e.s.d. in the dihedral angle between two l.s. planes) are estimated using the full covariance matrix. The cell e.s.d.'s are taken into account individually in the estimation of e.s.d.'s in distances, angles and torsion angles; correlations between e.s.d.'s in cell parameters are only used when they are defined by crystal symmetry. An approximate (isotropic) treatment of cell e.s.d.'s is used for estimating e.s.d.'s involving l.s. planes.
Refinement. Refinement of *F*^2^ against ALL reflections. The weighted *R*-factor *wR* and goodness of fit *S* are based on *F*^2^, conventional *R*-factors *R* are based on *F*, with *F* set to zero for negative *F*^2^. The threshold expression of *F*^2^ > σ(*F*^2^) is used only for calculating *R*-factors(gt) *etc*. and is not relevant to the choice of reflections for refinement. *R*-factors based on *F*^2^ are statistically about twice as large as those based on *F*, and *R*- factors based on ALL data will be even larger.

### Fractional atomic coordinates and isotropic or equivalent isotropic displacement parameters (Å^2^)

**Table:**

	*x*	*y*	*z*	*U*_iso_*/*U*_eq_	
N1	0.4602 (3)	0.40859 (16)	0.1386 (3)	0.0442 (7)	
N2	0.3606 (3)	0.34194 (16)	0.0989 (3)	0.0419 (7)	
O1	1.0054 (2)	0.64891 (13)	0.3680 (3)	0.0577 (7)	
H1A	0.9704	0.6998	0.3614	0.087*	
O2	0.6482 (2)	0.30744 (13)	0.1654 (2)	0.0521 (7)	
O3	−0.2256 (3)	0.12672 (15)	−0.0998 (3)	0.0675 (8)	
H3	−0.3047	0.1529	−0.1200	0.101*	
O4	−0.3830 (3)	0.26935 (19)	−0.1553 (3)	0.0766 (9)	
O5	−0.2875 (3)	0.40196 (19)	−0.1256 (3)	0.0801 (9)	
C1	0.7049 (3)	0.45812 (18)	0.2226 (3)	0.0344 (7)	
C2	0.6644 (3)	0.54841 (18)	0.2232 (3)	0.0411 (8)	
H2	0.5691	0.5655	0.1909	0.049*	
C3	0.7623 (3)	0.61274 (19)	0.2707 (3)	0.0432 (8)	
H3A	0.7332	0.6728	0.2699	0.052*	
C4	0.9033 (3)	0.58892 (19)	0.3194 (3)	0.0398 (8)	
C5	0.9456 (4)	0.49917 (19)	0.3202 (3)	0.0503 (10)	
H5	1.0406	0.4824	0.3534	0.060*	
C6	0.8471 (3)	0.4349 (2)	0.2720 (3)	0.0503 (10)	
H6	0.8766	0.3749	0.2726	0.060*	
C7	0.6036 (3)	0.38575 (19)	0.1743 (3)	0.0382 (8)	
C8	0.2280 (4)	0.3678 (2)	0.0654 (3)	0.0444 (9)	
H8	0.2047	0.4287	0.0685	0.053*	
C9	0.1110 (3)	0.3044 (2)	0.0222 (3)	0.0398 (8)	
C10	−0.0244 (3)	0.3372 (2)	−0.0261 (3)	0.0428 (8)	
H10	−0.0398	0.3989	−0.0301	0.051*	
C11	−0.1385 (3)	0.2798 (2)	−0.0692 (3)	0.0410 (8)	
C12	−0.1193 (4)	0.1874 (2)	−0.0631 (3)	0.0455 (9)	
C13	0.0193 (4)	0.1544 (2)	−0.0147 (3)	0.0486 (9)	
H13	0.0347	0.0926	−0.0102	0.058*	
C14	0.1324 (4)	0.2110 (2)	0.0262 (3)	0.0446 (9)	
H14	0.2243	0.1875	0.0569	0.053*	
N3	−0.2779 (3)	0.3202 (2)	−0.1195 (3)	0.0561 (8)	
H1	0.424 (3)	0.4649 (10)	0.144 (3)	0.067*	

### Atomic displacement parameters (Å^2^)

**Table:**

	*U*^11^	*U*^22^	*U*^33^	*U*^12^	*U*^13^	*U*^23^
N1	0.0310 (16)	0.0268 (13)	0.075 (2)	0.0000 (12)	−0.0032 (14)	−0.0041 (14)
N2	0.0364 (17)	0.0336 (14)	0.0558 (19)	−0.0036 (12)	0.0034 (14)	−0.0015 (13)
O1	0.0463 (15)	0.0301 (12)	0.0962 (19)	−0.0006 (11)	−0.0149 (14)	−0.0028 (13)
O2	0.0450 (14)	0.0267 (11)	0.0845 (18)	0.0024 (10)	−0.0015 (12)	−0.0041 (12)
O3	0.0593 (17)	0.0626 (16)	0.081 (2)	−0.0226 (13)	−0.0018 (15)	−0.0094 (15)
O4	0.0474 (17)	0.094 (2)	0.088 (2)	−0.0095 (16)	−0.0138 (15)	−0.0119 (17)
O5	0.0598 (18)	0.0628 (18)	0.117 (2)	0.0151 (15)	−0.0165 (16)	0.0017 (17)
C1	0.0307 (18)	0.0281 (15)	0.045 (2)	−0.0001 (13)	0.0075 (15)	0.0008 (14)
C2	0.033 (2)	0.0310 (17)	0.060 (2)	0.0037 (14)	0.0008 (17)	0.0031 (15)
C3	0.040 (2)	0.0259 (16)	0.064 (2)	0.0021 (14)	0.0000 (17)	0.0058 (16)
C4	0.0373 (19)	0.0296 (16)	0.052 (2)	−0.0024 (14)	0.0029 (17)	−0.0002 (15)
C5	0.036 (2)	0.0318 (17)	0.082 (3)	0.0063 (16)	−0.0109 (19)	−0.0018 (18)
C6	0.043 (2)	0.0273 (17)	0.080 (3)	0.0083 (15)	−0.004 (2)	−0.0029 (17)
C7	0.037 (2)	0.0315 (17)	0.046 (2)	0.0021 (14)	0.0081 (16)	0.0033 (15)
C8	0.041 (2)	0.0315 (17)	0.061 (2)	0.0032 (15)	0.0027 (18)	0.0009 (16)
C9	0.0335 (19)	0.0374 (17)	0.049 (2)	−0.0010 (14)	0.0075 (16)	0.0014 (16)
C10	0.037 (2)	0.0374 (18)	0.054 (2)	0.0014 (15)	0.0057 (17)	−0.0008 (16)
C11	0.034 (2)	0.048 (2)	0.042 (2)	0.0007 (15)	0.0065 (16)	−0.0042 (16)
C12	0.044 (2)	0.050 (2)	0.042 (2)	−0.0134 (17)	0.0061 (17)	−0.0068 (17)
C13	0.055 (2)	0.0335 (17)	0.057 (2)	−0.0027 (17)	0.0083 (19)	−0.0020 (17)
C14	0.041 (2)	0.0375 (19)	0.056 (2)	−0.0010 (15)	0.0033 (17)	0.0012 (16)
N3	0.0403 (19)	0.071 (2)	0.057 (2)	0.0008 (17)	0.0035 (16)	−0.0043 (17)

### Geometric parameters (Å, °)

**Table:**

N1—C7	1.349 (4)	C3—H3A	0.9300
N1—N2	1.380 (3)	C4—C5	1.387 (4)
N1—H1	0.899 (10)	C5—C6	1.376 (4)
N2—C8	1.269 (4)	C5—H5	0.9300
O1—C4	1.355 (3)	C6—H6	0.9300
O1—H1A	0.8200	C8—C9	1.458 (4)
O2—C7	1.234 (3)	C8—H8	0.9300
O3—C12	1.347 (4)	C9—C10	1.371 (4)
O3—H3	0.8200	C9—C14	1.403 (4)
O4—N3	1.244 (3)	C10—C11	1.383 (4)
O5—N3	1.222 (4)	C10—H10	0.9300
C1—C6	1.383 (4)	C11—C12	1.387 (4)
C1—C2	1.390 (4)	C11—N3	1.451 (4)
C1—C7	1.478 (4)	C12—C13	1.396 (4)
C2—C3	1.371 (4)	C13—C14	1.363 (4)
C2—H2	0.9300	C13—H13	0.9300
C3—C4	1.374 (4)	C14—H14	0.9300
			
C7—N1—N2	118.9 (2)	N1—C7—C1	117.5 (3)
C7—N1—H1	123 (2)	N2—C8—C9	121.7 (3)
N2—N1—H1	118 (2)	N2—C8—H8	119.2
C8—N2—N1	115.9 (2)	C9—C8—H8	119.2
C4—O1—H1A	109.5	C10—C9—C14	118.5 (3)
C12—O3—H3	109.5	C10—C9—C8	118.9 (3)
C6—C1—C2	118.0 (3)	C14—C9—C8	122.6 (3)
C6—C1—C7	118.4 (3)	C9—C10—C11	121.0 (3)
C2—C1—C7	123.6 (3)	C9—C10—H10	119.5
C3—C2—C1	121.1 (3)	C11—C10—H10	119.5
C3—C2—H2	119.4	C10—C11—C12	120.8 (3)
C1—C2—H2	119.4	C10—C11—N3	117.4 (3)
C2—C3—C4	120.4 (3)	C12—C11—N3	121.9 (3)
C2—C3—H3A	119.8	O3—C12—C11	124.7 (3)
C4—C3—H3A	119.8	O3—C12—C13	117.3 (3)
O1—C4—C3	123.4 (3)	C11—C12—C13	118.0 (3)
O1—C4—C5	117.2 (3)	C14—C13—C12	121.2 (3)
C3—C4—C5	119.4 (3)	C14—C13—H13	119.4
C6—C5—C4	120.0 (3)	C12—C13—H13	119.4
C6—C5—H5	120.0	C13—C14—C9	120.5 (3)
C4—C5—H5	120.0	C13—C14—H14	119.8
C5—C6—C1	121.1 (3)	C9—C14—H14	119.8
C5—C6—H6	119.4	O5—N3—O4	122.7 (3)
C1—C6—H6	119.4	O5—N3—C11	119.2 (3)
O2—C7—N1	120.9 (3)	O4—N3—C11	118.1 (3)
O2—C7—C1	121.6 (3)		

### Hydrogen-bond geometry (Å, °)

**Table:**

*D*—H···*A*	*D*—H	H···*A*	*D*···*A*	*D*—H···*A*
O1—H1A···O2^i^	0.82	1.93	2.736 (3)	169
O1—H1A···N2^i^	0.82	2.61	3.121 (3)	122
O3—H3···O4	0.82	1.90	2.592 (4)	142
N1—H1···O5^ii^	0.90 (1)	2.32 (1)	3.203 (4)	167 (3)

Symmetry codes: (i) −*x*+3/2, *y*+1/2, −*z*+1/2; (ii) −*x*, −*y*+1, −*z*.
